# Effect of Chronic Obstructive Pulmonary Disease (COPD) on Biventricular Mechanics in Patients Without Severe Airflow Obstruction

**DOI:** 10.3390/jcm14113660

**Published:** 2025-05-23

**Authors:** Andrea Sonaglioni, Massimo Baravelli, Antonella Caminati, Federico Tagariello, Federico De Cesco, Gian Luigi Nicolosi, Michele Lombardo, Sergio Harari

**Affiliations:** 1 Division of Cardiology, IRCCS MultiMedica, 20123 Milan, Italy; massimo.baravelli@multimedica.it (M.B.); michele.lombardo@multimedica.it (M.L.); 2 Division of Pneumology, Semi-Intensive Care Unit, IRCCS MultiMedica, 20123 Milan, Italy; antonella.caminati@multimedica.it (A.C.); federico.tagariello@multimedica.it (F.T.); federico.decesco.m@gmail.com (F.D.C.); sergio@sergioharari.it (S.H.); 3 Division of Cardiology, Policlinico San Giorgio, 33170 Pordenone, Italy; gianluigi.nicolosi@gmail.com; 4 Department of Clinical Sciences and Community Health, Università di Milano, 20122 Milan, Italy

**Keywords:** chronic obstructive pulmonary disease, subclinical myocardial dysfunction, biventricular mechanics, LV-GLS, RV-GLS

## Abstract

**Background:** Over the last 15 years, few echocardiographic studies have examined the biventricular mechanics by speckle tracking echocardiography (STE) in patients affected by chronic obstructive pulmonary disease (COPD) without advanced lung disease. We aimed to summarize the main findings of these studies and quantify the overall effect of COPD on biventricular mechanics in patients without severe airflow obstruction. **Methods:** Eligible studies assessing cardiac function by conventional transthoracic echocardiography (TTE), implemented with a STE analysis of left ventricular (LV)-global longitudinal strain (GLS) and/or right ventricular (RV)-GLS in COPD patients without severe airflow obstruction vs. healthy controls, were selected from the PubMed, Embase and Scopus databases. The primary endpoint was to quantify the effect of COPD on LV-GLS and RV-GLS in individuals without advanced lung disease. Continuous data [LV-GLS, RV-GLS, left ventricular ejection fraction (LVEF) and tricuspid annular plane systolic excursion (TAPSE)] were pooled as the standardized mean difference (SMD) comparing COPD cohorts with healthy controls. **Results:** Ten studies were included, totaling 682 COPD patients and 316 healthy controls. Overall, COPD showed a large effect on LV-GLS (SMD −1.296; 95%CI −2.010, −0.582, *p* < 0.001) and RV-GLS (SMD −1.474; 95% CI −2.142, −0.805, *p* < 0.001), a medium-to-large effect on TAPSE (SMD −0.783, 95% CI −0.949, −0.618, *p* < 0.001) and a small effect on LVEF (SMD −0.366, 95% CI −0.659, −0.074, *p* = 0.014). The I^2^ statistic value for the LV-GLS (91.1%), RV-GLS (88.2%) and LVEF (76.7%) studies suggested a high between-study heterogeneity, while that for the TAPSE (38.1%) studies was compatible with a low-to-moderate between-study heterogeneity. Egger’s test yielded a *p*-value of 0.16, 0.48, 0.58 and 0.50 for LV-GLS, RV-GLS, LVEF and TAPSE studies, respectively, indicating an absence of publication bias. Meta-regression analyses excluded that the effect of COPD on biventricular mechanics might be influenced by potential confounders (all *p* > 0.05). Sensitivity analysis confirmed the robustness of the LV-GLS, RV-GLS and TAPSE studies’ results. **Conclusions:** COPD appears to be independently associated with a mild attenuation of biventricular mechanics in patients with moderate airflow limitations, despite a preserved LVEF and TAPSE on conventional TTE. STE analysis may allow clinicians to identify COPD patients with subclinical myocardial dysfunction and an increased risk of heart failure and cardiovascular complications early.

## 1. Introduction

Chronic obstructive pulmonary disease (COPD) is a heterogenous lung condition characterized by chronic respiratory symptoms (dyspnea, cough and sputum production) due to abnormalities of the airways (bronchitis, bronchiolitis) and/or alveoli (emphysema) that cause persistent and often progressive airflow obstruction [1]. Its prevalence has rapidly increased during the last decades, affecting over 400 million people globally [2].

COPD is frequently associated with several cardiovascular comorbidities, especially pulmonary hypertension (PH), cardiac arrhythmias and coronary artery disease (CAD) [3,4,5]. The increased cardiovascular disease burden of COPD patients represents a leading cause of morbidity and mortality in these patients [6].

In clinical practice, most COPD patients are referred to outpatient cardiology clinics by pulmonologists or internal medicine physicians to perform conventional transthoracic echocardiography (TTE) for the assessment of pulmonary hemodynamics. It is noteworthy that severe PH and right ventricular (RV) systolic dysfunction are usually detectable by TTE only in patients with advanced COPD [7]. However, cardiovascular risk factors and comorbidities may affect cardiac function even in mild-to-moderate COPD, without affecting global cardiac contractility, which is assessed by left ventricular ejection fraction (LVEF). For this reason, in recent years, researchers have focused their attention on the identification of early markers of myocardial systolic function that could be more sensitive than LVEF in detecting subclinical myocardial organ damage.

Speckle tracking echocardiography (STE) is an innovative angle-independent imaging modality that evaluates the myocardial deformation properties of cardiac chambers throughout the cardiac cycle. It allows for measuring the deformation (“strain”) of both ventricles and both atria and the rate at which this deformation occurs (“strain rate”) [8]. STE analysis is able to provide incremental diagnostic and prognostic information over traditional TTE, as consistently demonstrated in various clinical settings [9,10,11]. Despite its potential, the STE methodology has been rarely applied for evaluating the cardiac function in COPD patients. Indeed, over the last 15-year period, only a few echocardiographic studies have accurately examined the biventricular mechanics by TTE implemented with STE in COPD patients. These studies aimed at identifying an early STE marker of subclinical myocardial dysfunction in the presence of preserved LVEF on TTE, in COPD patients without severe airflow obstruction. However, most of these studies were designed to evaluate the COPD influence on left ventricular (LV) or RV mechanics separately, whereas a concomitant assessment of the biventricular mechanics in non-advanced COPD was rarely provided. The present systematic review and meta-analysis aimed to summarize the principal findings of these studies and to quantify the overall effect of COPD on biventricular mechanics in patients without advanced lung disease. The pathophysiological mechanisms underpinning subclinical myocardial dysfunction in COPD patients will be discussed as well.

## 2. Materials and Methods

This systematic review and meta-analysis were performed according to the PRISMA guidelines [12] and were registered in INPLASY (registration number INPLASY202540086).

### 2.1. Search Strategy

Two reviewers (A.S. and M.B.) independently accessed the PubMed, Scopus, Embase, Cochrane and Web of Science databases to research all echocardiographic studies that, regardless of the timeframe, performed conventional TTE implemented with a STE analysis of the biventricular mechanics in COPD patients without severe airflow limitation (between 10 April 2025 and 17 April 2025). The search strategy included the following terms: “chronic obstructive pulmonary disease”, “COPD”, “cardiac function”, “biventricular mechanics”, “left ventricular mechanics”, “left ventricular global longitudinal strain”, “LV-GLS”, “right ventricular mechanics”, “right ventricular global longitudinal strain”, “RV-GLS”. No language restrictions were imposed.

### 2.2. Eligibility Criteria

All case-control studies assessing cardiac function by traditional TTE implemented with STE analysis of the biventricular mechanics in hemodynamically stable COPD patients in the Global Initiative for Chronic Obstructive Lung Disease (GOLD) stage I, II or III [defined by the forced expiratory volume in the first second (FEV1)/forced vital capacity (FVC) ratio < 70% and FEV1 ≥ 80% (stage I), between 50% and 79% (stage II), and between 30% and 49% (stage III) of the predicted values, respectively] [13] vs. healthy individuals without COPD were included in this systematic review and meta-analysis. The criteria of exclusion were as follows: echocardiographic studies that focused on COPD patients in the GOLD stage IV (defined by a FEV1/FVC ratio < 70% and FEV1 < 30% of the predicted values) [13]; echocardiographic studies that analyzed COPD patients with severe PH [14], hemodynamic instability, acute exacerbation of COPD and/or congestive right heart failure (RHF); imaging studies that performed TTE in COPD patients without a concomitant assessment of the myocardial strain parameters by STE; echocardiographic studies that analyzed COPD individuals without controls; nonechocardiographic studies; and nonclinical studies.

### 2.3. Study Selection and Data Extraction

Two reviewers (A.S. and M.B.) screened the records according to the aforementioned eligibility criteria and independently collected the following information concerning COPD patients and the controls: (1) demographics (age and sex); (2) anthropometrics [the body surface area (BSA) and body mass index (BMI); (3) prevalence of the principal cardiovascular risk factors (hypertension, smoking, type 2 diabetes and dyslipidemia); (4) COPD severity according to the GOLD classification [13] and/or the Body Mass Index, Airflow Obstruction, Dyspnea and Exercise (BODE) index [15]; (5) hemodynamics [the cardiac rhythm, heart rate, systolic blood pressure (SBP) and diastolic blood pressure (DBP)]; (6) pulmonary function tests (PFTs), including the FEV1, the FVC and the resulting FEV1/FVC ratio, and the residual volume (RV); (7) diffusion capacity of the lungs for carbon monoxide (DLCO) and the distance covered during a 6-Minute Walk Test (6MWT); (8) arterial blood gas (ABG) analysis results in room air; (9) relevant cardiovascular and noncardiovascular comorbidities; (10) blood tests that were comprehensive of the serum levels of hemoglobin, creatinine, fasting plasma glucose, low-density lipoprotein (LDL) cholesterol and N-terminal pro B-type natriuretic peptide (NT-proBNP); (11) traditional echoDoppler indices of cardiac chambers’ cavity sizes, biventricular systolic function and pulmonary hemodynamics; (12) STE-derived biventricular myocardial strain parameters; (13) and finally, current respiratory and nonrespiratory treatments.

### 2.4. Risk of Bias Assessment

The risk of bias (RoB) was assessed by using the National Institutes of Health (NIH)’s Quality Assessment of Case-Control Studies [16]. Two authors (A.S. and G.L.N.) independently estimated the quality rating of each study as “good”, “fair” or “poor”. Possible discrepancies between the investigators were resolved through a consensus discussion with the involvement of a third author (M.B.). Cohen’s kappa coefficient [17] was the statistical measure employed to quantify the level of agreement between the two raters.

### 2.5. Statistical Analysis

Continuous data were summarized as the median [(interquartile range (IQR)], whereas the categorical variables were reported as percentages (%). Assuming that the underlying distribution was normal or that it did not relevantly deviate from a normal distribution, the mean was considered equal to the median and, to estimate the standard deviation (SD) from the IQR, the following equation was used: SD = IQR/1.35.

The primary endpoint was to quantify the effect of COPD on LV-GLS and RV-GLS in individuals in GOLD stages I to III. Continuous data (LV-GLS, RV-GLS, LVEF and TAPSE) were pooled as the standardized mean difference (SMD), comparing the COPD cohorts with healthy controls. The overall SMDs of LV-GLS, RV-GLS and LVEF were calculated using the random-effect model due to the increased between-study heterogeneity. The I-squared statistic (I^2^) was used to quantify the percentage of variation across studies. Publication bias was assessed by using Begg’s funnel plots and Egger’s test. Meta-regression analysis was performed to determine whether the effect of COPD on biventricular mechanics might be influenced by potential confounders, in particular, age, BMI and SBP for LV-GLS, and the specific ultrasound machine was employed for the STE analysis and systolic pulmonary artery pressure (sPAP) for RV-GLS. Finally, sensitivity analyses were performed to explore the impact of removing each of the studies on the overall SMDs of LV-GLS, RV-GLS and TAPSE. A statistical analysis was performed with the software Comprehensive Meta-Analysis version 3.0 (Biostat, Englewood, NJ, USA), and two-tailed *p*-values below 0.05 were considered statistically significant.

## 3. Results

### 3.1. Study Selection

**Figure 1:**
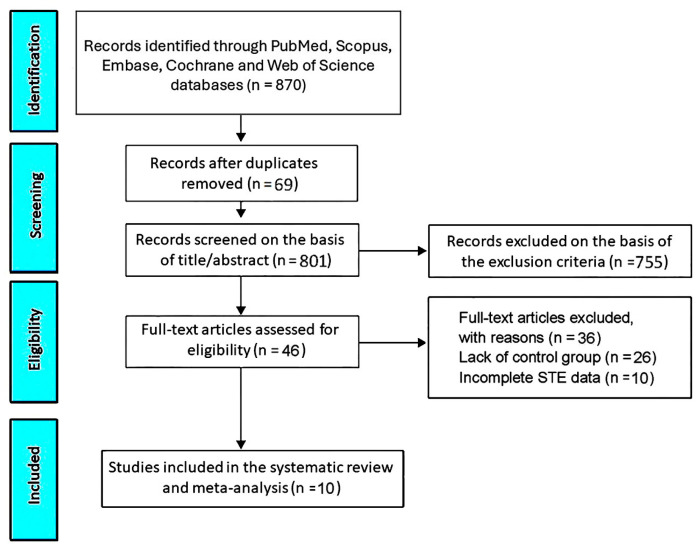
The PRISMA flow diagram used for identifying the included studies. STE, speckle tracking echocardiography.

By accessing the PubMed, Scopus, Embase, Cochrane and Web of Science databases, the initial research allowed us to identify 870 studies. Sixty-nine studies (7.9% of the total) were removed as duplicates. Based on the exclusion criteria, 755 studies (86.8% of the total) were excluded. The remaining 46 studies (5.3% of the total) were assessed for eligibility. Of those, 26 (3% of total) were excluded due to the lack of a control group and 10 (1.1% of total) due to incomplete STE data. Accordingly, 10 studies (1.1% of the total) [18,19,20,21,22,23,24,25,26,27] were included in this systematic review and meta-analysis (Figure 1), totaling 682 COPD patients and 316 healthy controls without COPD.

### 3.2. Clinical Findings

Table 1 summarizes the clinical characteristics and the most relevant echocardiographic findings of the studies included in this systematic review and meta-analysis.

The included studies were published between 2010 and 2025. Three studies were conducted by authors from Turkey, whereas the remaining seven studies were performed in the United Kingdom, Norway, Germany, China, India, the Netherlands and Vietnam. The median age of the COPD participants was 63.5 yrs (range 49.1–70 yrs), while the median age of the controls was 61.6 yrs (range 48.9–67 yrs). Approximately two-thirds of the COPD patients were males. Most of the included studies were prospective, whereas only the study of Goedemans L., et al. [25] had a retrospective design. Concerning the ultrasound system employed for STE analysis, five studies (50% of total) used a General Electric (GE) machine, four (40% of total) a Philips Software, while Kalaycıoğlu, E., et al. [20] did not specify the ultrasound machine used for measuring the myocardial strain parameters. Only two studies (20% of the total) provided follow-up data. Notably, Kanar B.G., et al. [23] found an improvement in RV mechanics in COPD patients who underwent a 4-week pulmonary rehabilitation (PR) program, while Nasir S.A., et al. [24] demonstrated a significant deterioration of RV function over a 6-month follow-up in approximately one-third of the COPD patients, particularly in those with baseline abnormal RV function and a sPAP ≥ 35 mmHg.

All the demographic, anthropometric, clinical, hemodynamic, spirometric and biochemical parameters collected at baseline by the included studies in COPD patients and healthy controls are listed in Table 2.

The most frequently assessed parameters were demographic, anthropometric, hemodynamic and spirometric, whereas information concerning the burden of cardiovascular risk factors and cardiovascular comorbidities, blood tests and the current medical treatment was provided by a limited number of studies, ranging from 10 and 50% of the total. Overall, the COPD participants were elderly males with a moderate prevalence of hypertension, current smoking and dyslipidemia and a low prevalence of type 2 diabetes. Among the cardiovascular comorbidities, assessed by only two studies [21,25], CAD history was detected in 39.1% of the patients (range 28.2–50%). Only Goedemans L. et al. [25] evaluated the prevalence of noncardiovascular comorbidities among COPD individuals, and, in particular, obstructive sleep apnea syndrome (OSAS) was detected in only 4% of them. An analysis of the hemodynamics showed atrial fibrillation (AF) in 10.1% of the patients; overall, the COPD patients were found with well-controlled blood pressure and a normal resting heart rate. Pulmonary function impairment, evaluated according to the GOLD classification, was moderate [median GOLD stage 1.9 (range 1.5–2.5)], with a median BODE index of 2.5 (range 2–3). Baseline PFTs confirmed a moderate airflow obstruction. A concomitant moderate reduction in DLCO and in effort tolerance, as assessed by the 6MWT, was detected. ABG revealed mild hypoxemia with a normal partial pressure of carbon dioxide in the arterial blood (PaCO2). In the blood tests, the COPD patients presented with mild renal failure (a median creatinine value of 0.97 mg/dL, corresponding to a median estimated glomerular filtration rate of 83 mL/min/m^2^) [28], mild hypercholesterolemia [a median LDL cholesterol value of 129.8 mg/dL (range 119.1–140.5 mg/dL)] and a moderate increase in serum levels of NT-proBNP [a median value of 268.4 pg/mL (range 83.7–453.2 pg/mL]. Compared to the healthy controls, the COPD individuals were slightly older, had significantly lower BMIs, had a significantly higher prevalence of the most relevant cardiovascular risk factors, a CAD history and concomitant AF, significantly higher resting heart rate and blood pressure values and, finally, significantly higher serum levels of creatinine, fasting glucose, LDL cholesterol and NT-proBNP (all *p* values < 0.05). Regarding medical therapy, the COPD patients were predominantly prescribed inhaled treatments, particularly long-acting beta2-agonists, long-acting anticholinergics and inhaled glucocorticoids. Conversely, cardioprotective drugs were prescribed to a smaller proportion of COPD patients, ranging from 4% to 45% of the total.

### 3.3. Traditional Echocardiography and Strain Imaging Findings

All the included studies were performed using conventional TTE, implemented with STE analyses of the LV and/or RV myocardial deformation indices (Table 3).

The most commonly measured TTE parameters were the LVEF, sPAP, TAPSE, E/e’ ratio and TDI-derived RV basal s’, while the great majority of traditional echocardiographic indices were assessed by only 20–40% of the studies. COPD patients were diagnosed with LV concentric remodeling, a preserved LVEF [median value 60% (range 50–67.8%)], an E/e’ ratio in the so-called “gray zone” between 8 and 13 [median value 9.7 (range 5.8–17)], mild left atrial (LA) enlargement, a normal RV size, preserved RV systolic function, as assessed by tricuspid annular plane systolic excursion (TAPSE) magnitude [median value 19.3 mm (range 16.6–23 mm)] and, finally, a normal sPAP [median value 32.3 mmHg (range 22.9–46.7 mmHg)]. Compared to the healthy controls, the COPD patients had significantly smaller LV end-diastolic internal dimensions, greater relative wall thicknesses, higher left ventricular filling pressures, assessed by the E/e’ ratio [29], lower LVEFs, larger LA and RV sizes, lower TAPSE and a higher sPAP.

The STE analysis of myocardial strain parameters was primarily focused on LV-GLS (assessed by 60% of studies) and RV-GLS (measured by 50% of studies), whereas the remaining STE indices were evaluated by a limited number of echocardiographic studies, ranging from 10 to 30% of the total. Only two studies [18,27] evaluated both LV and RV mechanics in COPD patients. The median values of LV-GLS, RV-GLS and RV-FWLS were significantly lower in the COPD patients vs. healthy controls, while LV-global longitudinal strain rate in systole (GLSRs), LV-global circumferential strain (GCS) and LV-global radial strain (GRS) were not statistically different in the two study groups. Even if biventricular mechanics resulted in being significantly impaired in the COPD participants vs. healthy controls, when considering the accepted reference values for both the LV [30] and RV [31] myocardial deformation indices, the magnitude of attenuation of LV-GLS, RV-GLS and RV-FWLS detected in COPD patients was overall mild. Differently from the other authors, Pizarro, C. et al. [21] performed a detailed analysis of regional LV strain in COPD participants, demonstrating a localized deterioration of apical septal longitudinal strain. A concomitant assessment of biatrial mechanics was performed by two studies (20% of total); Goedemans L. et al. [25] described a significant deterioration of both left atrial reservoir strain (LASr) and right atrial reservoir strain (RASr) in COPD patients with AF, while Nguyen Ngoc Dang H. et al. [27] did not find a statistically significant difference in the LASr magnitude between COPD patients and healthy controls.

**Figure 2:**
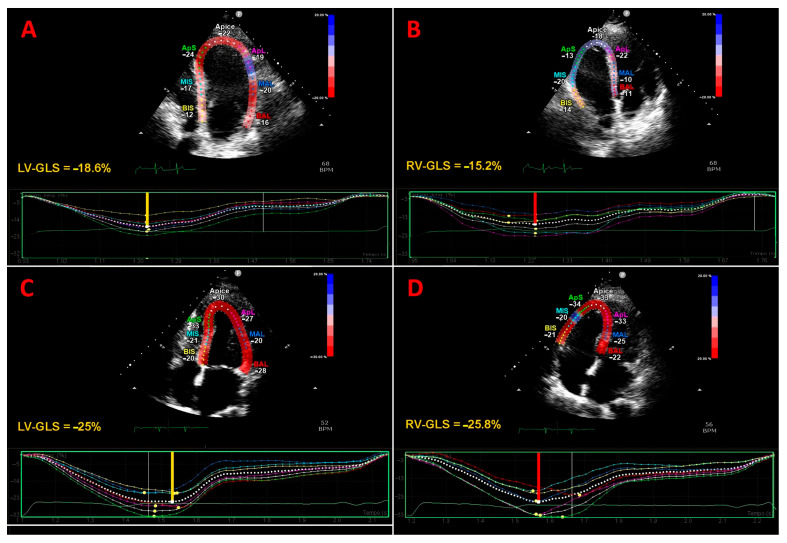
Representative examples of LV-GLS and RV-GLS assessments from the apical four-chamber view in a COPD patient (**A**,**B**) and in a healthy control without COPD (**C**,**D**). The bold yellow line and red line indicate the LV-GLS and RV-GLS magnitudes, respectively, obtained in the two individuals. COPD, chronic obstructive pulmonary disease; GLS, global longitudinal strain; LV, left ventricular; RV, right ventricular.

Figure 2 depicts representative examples of the LV-GLS and RV-GLS assessments from the apical four-chamber view in a COPD patient (Figure 2A,B) and in a healthy control without COPD (Figure 2C,D).

### 3.4. NIH Quality Rating

The NIH quality rating was estimated as good for four studies and fair for six studies (Table 4).

The estimated Cohen’s kappa coefficient k was 0.81, compatible with substantial agreement between the reviewers in the RoB assessment.

### 3.5. Effect of COPD on LV-GLS

**Figure 3:**
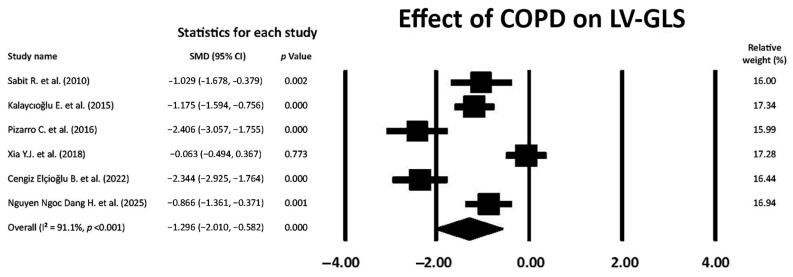
Forest plot showing the effect of COPD on LV-GLS across the included studies [18,20,21,22,26,27]. COPD, chronic obstructive pulmonary disease; GLS, global longitudinal strain; LV, left ventricular.

COPD showed a large effect on LV-GLS magnitude (SMD −1.296; 95% CI −2.010, −0.582, *p* < 0.001), thus indicating the early occurrence of LV myocardial deformation impairment in the longitudinal direction in COPD patients without severe airflow limitation (Figure 3).

The overall I^2^ statistic value was 91.1%, suggesting high between-study heterogeneity. Begg and Mazumdar’s test for rank correlation gave a *p*-value of 0.35, indicating no evidence of publication bias. Egger’s test produced a *p-*value of 0.16, indicating no evidence of publication bias.

**Figure 4:**
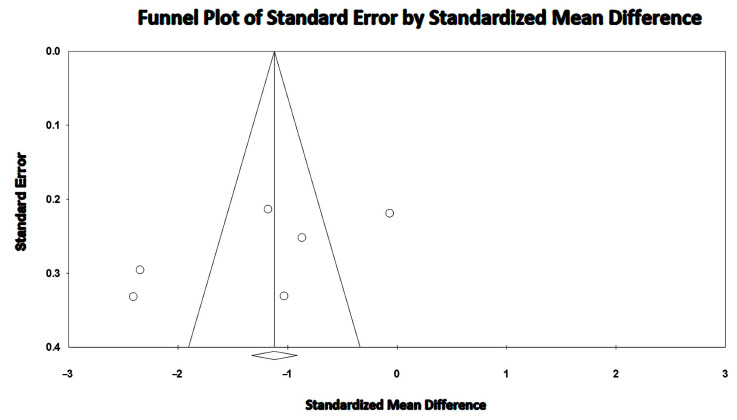
Begg’s funnel plot for the detection of publication bias in LV-GLS studies. GLS, global longitudinal strain; LV, left ventricular.

The Begg’s funnel plot for LV-GLS studies is illustrated in Figure 4.

The meta-regression analysis allowed the exclusion of any correlation between potential confounders (age, BMI and SBP) and LV-GLS in COPD patients (all *p* > 0.05) (Table 5).

The sensitivity analysis confirmed the robustness of the study’s results on LV-GLS assessment. The sequential removal of each study caused a mild variability in SMD, from −1.132 (95% CI −1.484, −0.780, *p* < 0.001) to −1.520 (95% CI −2.307, −0.733, *p* < 0.001).

### 3.6. Effect of COPD on RV-GLS

**Figure 5:**
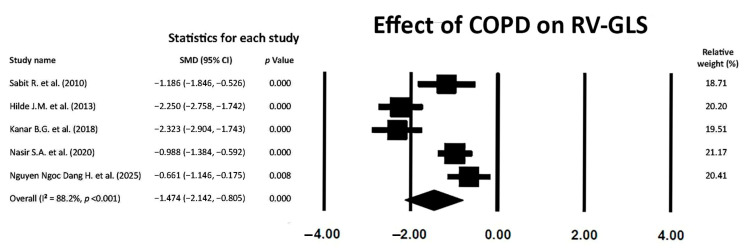
Forest plot showing the effect of COPD on RV-GLS across the included studies [18,19,23,24,27]. COPD, chronic obstructive pulmonary disease; GLS, global longitudinal strain; RV, right ventricular.

COPD also exerted a large effect on RV-GLS magnitude (SMD −1.474; 95% CI −2.142, −0.805, *p* < 0.001), thus indicating the early occurrence of RV myocardial deformation impairment in the longitudinal direction in COPD patients without severe airflow limitation (Figure 5).

A high between-study heterogeneity was detected (I^2^ statistic value 88.2%, *p* < 0.001). Begg and Mazumdar’s test for rank correlation provided a *p*-value of 0.62, indicating no evidence of publication bias. Egger’s test gave a *p-*value of 0.48, thus excluding publication bias.

**Figure 6:**
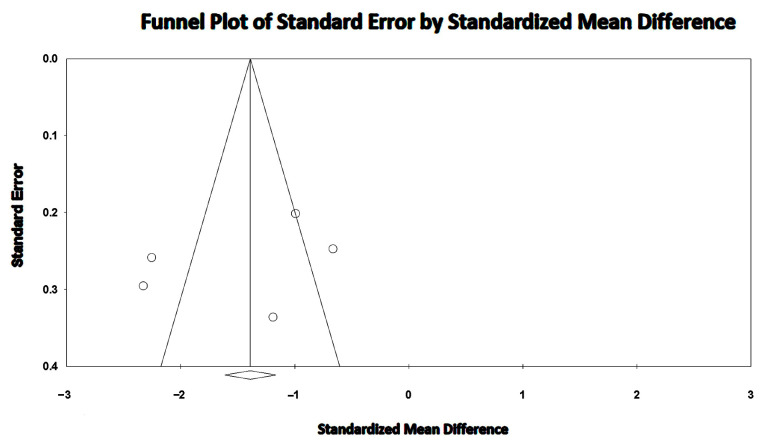
Begg’s funnel plot for the detection of publication bias in RV-GLS studies. GLS, global longitudinal strain; RV, right ventricular.

The Begg’s funnel plot for RV-GLS studies is depicted in Figure 6.

The meta-regression analysis excluded any correlation between potential confounders (the ultrasound machine employed for STE analysis and sPAP) and RV-GLS in COPD patients (all *p* > 0.05) (Table 6).

The sensitivity analysis confirmed the robustness of the study’s results on RV-GLS assessment. The sequential removal of each study caused a mild variability in SMD, from −1.682 (95% CI −2.407, −0.957, *p* < 0.001) to −1.944 (95% CI −2.612, −1.275, *p* < 0.001).

### 3.7. Effect of COPD on LVEF

**Figure 7:**
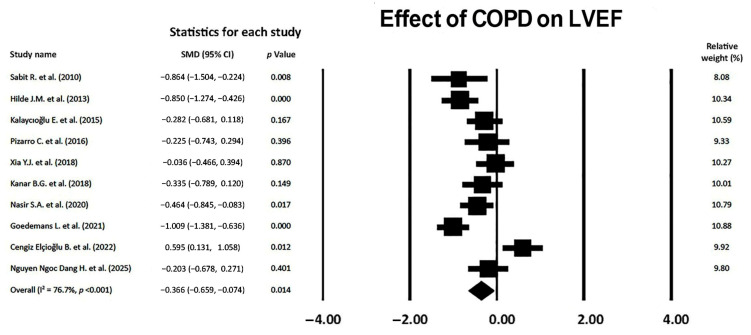
Forest plot showing the effect of COPD on LVEF across the included studies [18,19,20,21,22,23,24,25,26,27]. COPD, chronic obstructive pulmonary disease; LVEF, left ventricular ejection fraction.

Overall, COPD showed a small effect on LVEF magnitude (SMD −0.366, 95% CI −0.659, −0.074, *p* = 0.014), thus excluding a LVEF reduction during the early stages of COPD (Figure 7).

The between-study heterogeneity was high (I^2^ statistic value 76.7%, *p* < 0.001). Begg and Mazumdar’s test for rank correlation gave a *p*-value of 0.24, indicating no evidence of publication bias. Egger’s test yielded a *p-*value of 0.58, indicating the absence of publication bias.

**Figure 8:**
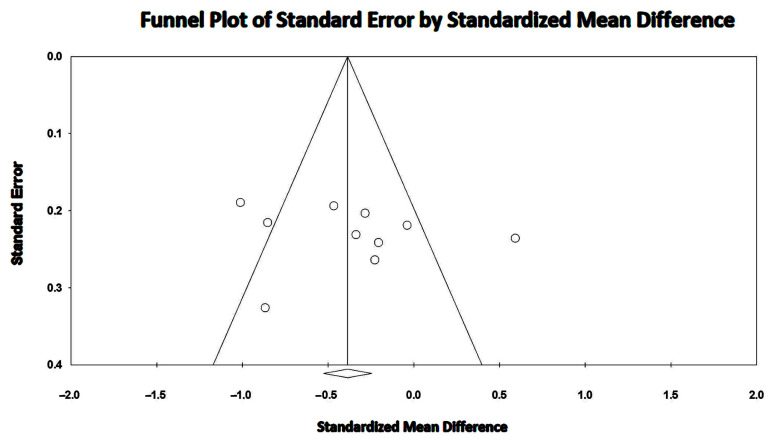
Begg’s funnel plot for the detection of publication bias in LVEF studies. LVEF, left ventricular ejection fraction.

The Begg’s funnel plot for LVEF studies is shown in Figure 8.

### 3.8. Effect of COPD on TAPSE

**Figure 9:**
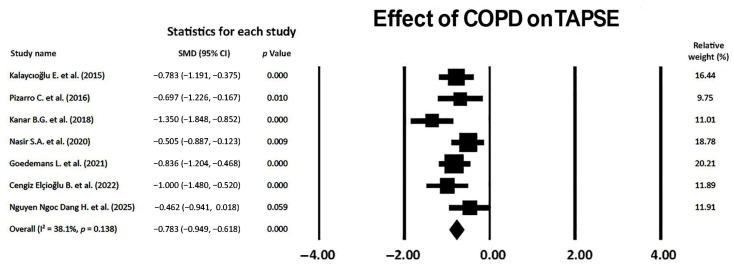
Forest plot showing the effect of COPD on TAPSE across the included studies [20,21,23,24,25,26,27]. COPD, chronic obstructive pulmonary disease; TAPSE, tricuspid annular plane systolic excursion.

A medium-to-large effect of COPD on TAPSE magnitude was observed (SMD −0.783, 95% CI −0.949, −0.618, *p* < 0.001), thus indicating a moderate effect of non-advanced COPD on RV longitudinal systolic function (Figure 9).

The I^2^ statistic value was 38.1% (*p* = 0.138), compatible with a low-to-moderate between-study heterogeneity. Begg and Mazumdar’s test for the rank correlation gave a *p*-value of 0.45, indicating no evidence of publication bias. Egger’s test gave a *p-*value of 0.50, thus excluding the presence of publication bias.

**Figure 10:**
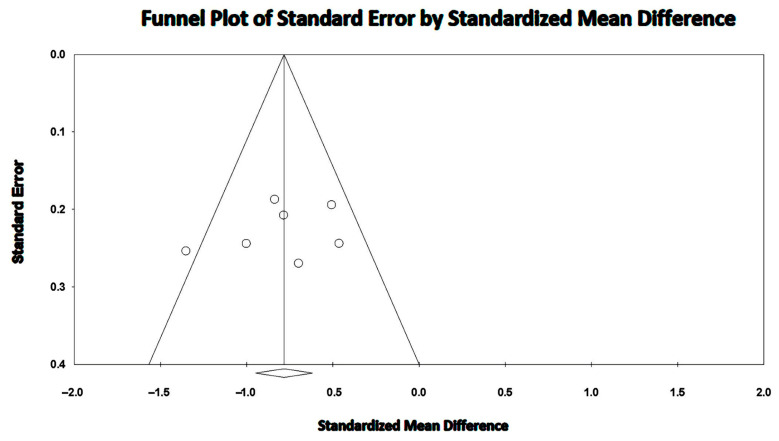
Begg’s funnel plot for the detection of publication bias in the TAPSE studies. TAPSE, tricuspid annular plane systolic excursion.

The Begg’s funnel plot for the TAPSE studies is illustrated in Figure 10.

The sensitivity analysis confirmed the robustness of the study’s results on the TAPSE assessment. The sequential omission of each study caused a mild variability in SMD, from −0.751 (95% CI −1.074, −0.428, *p* < 0.001) to −0.928 (95% CI −1.199, −0.657, *p* < 0.001).

## 4. Discussion

### 4.1. Main Findings

This systematic review and meta-analysis, analyzing 10 case-control studies conducted over a 15-year period, including 682 COPD patients, demonstrated that COPD participants (1) were predominantly elderly males with a moderate prevalence of hypertension, smoking, dyslipidemia and a low prevalence of type 2 diabetes; (2) were affected by moderate airflow limitation, as assessed by the GOLD classification and PFTs results; (3) were found with good blood pressure control but suboptimal control of LDL cholesterol levels; (4) on conventional TTE, were diagnosed with mild LV concentric remodeling, preserved biventricular systolic function (assessed by LVEF and TAPSE, respectively), subclinical diastolic dysfunction and normal hemodynamics (median sPAP < 35 mmHg); (5) on strain echocardiographic imaging, were found with a significant, although modest, attenuation of biventricular mechanics (expressed by LV-GLS and RV-GLS magnitude, respectively) compared to healthy controls and to the accepted reference values [30,31]; (6) received adequate respiratory treatment, but were, overall, underprescribed with cardioprotective drugs.

Our meta-analysis revealed that COPD had a large effect on LV-GLS and RV-GLS, a medium-to-large effect on TAPSE and a small effect on LVEF. The effect of COPD on biventricular mechanics was independent of the relevant moderators, as demonstrated by the meta-regression analyses conducted separately for LV-GLS and RV-GLS. Notably, COPD showed a negative influence on LV mechanics independently of age, anthropometrics and blood pressure values; additionally, the RV myocardial deformation properties resulted in impairment, regardless of the ultrasound machine employed for STE analysis and pulmonary hemodynamics.

The high between-study heterogeneity detected for the imaging studies assessing LV-GLS, RV-GLS and LVEF in COPD patients was likely related to technical factors, such as the intervendor variability in the STE examination of biventricular mechanics and the possible inclusion of COPD patients affected by various degrees of lung disease, as assessed by GOLD classification.

The results of this meta-analysis would suggest the early occurrence of specific cardiac remodeling in COPD patients with moderate airflow obstruction, characterized by subclinical LV diastolic dysfunction and a mild attenuation of biventricular mechanics on STE analysis in the presence of preserved biventricular systolic function on conventional TTE.

### 4.2. Pathophysiological Mechanisms Underpinning the Early Deterioration of Biventricular Mechanics in COPD Patients

**Figure 11:**
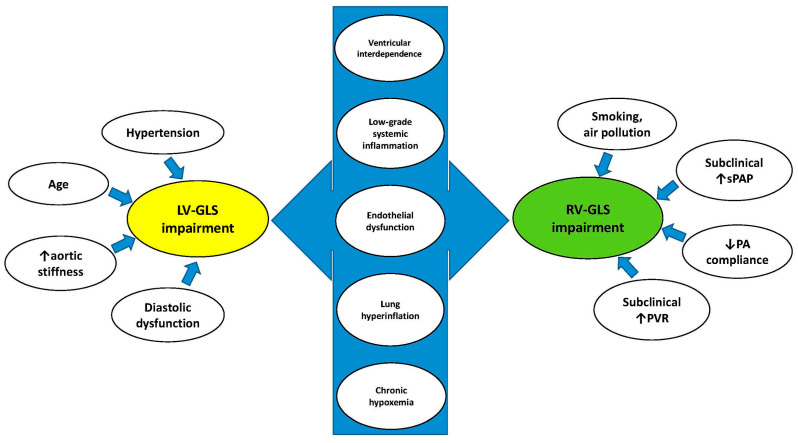
Pathophysiological mechanisms underpinning the early deterioration of biventricular mechanics in COPD patients. COPD, chronic obstructive pulmonary disease; GLS, global longitudinal strain; LV, left ventricular; PVR, pulmonary vascular resistance; RV, right ventricular; sPAP, systolic pulmonary artery pressure.

Several pathophysiological mechanisms might explain the early occurrence of subclinical biventricular systolic dysfunction in COPD patients (Figure 11).

Concerning LV-GLS impairment, it is known that aging [32] and hypertension [33] are associated with greater LV concentricity, impaired myocardial relaxation and reduced LV-GLS. Additionally, LV-GLS may be impaired due to abnormalities in LV afterload, related to increased aortic stiffness [18]. All these factors may synergically cause subclinical diastolic dysfunction in COPD patients without advanced lung disease, leading to myocardial fibrosis with consequent LV-GLS deterioration.

The early alteration in RV mechanics observed in COPD patients has been attributed to the harmful effects of smoking [34] and/or air pollution [35], to a slight and subclinical increase in sPAP [36], pulmonary vascular resistance (PVR) [19], pulmonary artery stiffness [37] and/or to the possible transient increase in RV afterload occurring during the daily life activities of these individuals [38]. In COPD patients without advanced lung disease, a normal RV–pulmonary artery (PA) coupling allows the right ventricle to effectively contract and eject blood into the pulmonary arteries, matching the afterload [39]. During the initial stages of lung disease, COPD causes an early subclinical attenuation in RV mechanics, expressed by RV-GLS reduction and assessed by STE analysis; subsequently, PH development and worsening are associated with gradual TAPSE deterioration, leading to RV–PA uncoupling and, ultimately, right heart failure [40].

Additive mechanisms might have a negative impact on both LV-GLS and RV-GLS in COPD individuals. Considering that the left ventricle and the right ventricle share the interventricular septum (ventricular interdependence), even a mild enlargement of the right ventricle may cause not only a subclinical decline in RV mechanics but may also cause LV diastolic dysfunction and the subclinical impairment in LV mechanics [19,26].

Another potential mechanism underlying the concomitant reduction in LV and RV myocardial function is the presence of persistent low-grade systemic inflammation, consistently reported in COPD individuals [41,42,43]. Chronic inflammation is associated with blood leukocytosis, increased serum levels of C-reactive protein, fibrinogen and inflammatory cytokines. Systemic inflammation occurring in COPD patients may contribute to the development of both pulmonary and systemic endothelial dysfunction, thus accelerating biventricular functional deterioration and heart failure occurrence [44,45,46].

In addition, by reducing cardiac preload, lung hyperinflation secondary to emphysema may affect LV filling and cause a reduction in stroke volume and cardiac output, even in the presence of preserved LVEF. Moreover, the increased intrathoracic pressure from hyperinflation can compress both the right-sided and left-sided cardiac chamber cavity sizes, thus impairing biventricular mechanics. It is noteworthy that these intrathoracic compressive phenomena may be associated with extrinsic compressive phenomena on cardiac chambers, as demonstrated by our study group in non-COPD cohorts with android obesity [47] or pectus excavatum [48].

Finally, chronic hypoxemia, a common finding in COPD individuals, has been associated with cardiac coronary artery and venous ischemia affecting LV mechanics [49], and with hypoxic vasoconstriction causing pulmonary hypertension, thus affecting RV mechanics [50].

### 4.3. Implications for Clinical Practice

The included studies demonstrated a strong correlation between COPD severity, assessed by the GOLD classification and/or BODE index and/or PFTs, and the degree of biventricular mechanics impairment. As demonstrated by the two studies that provided short-term follow-up data [23,24], COPD patients may experience a rapid deterioration of clinical, respiratory and echocardiographic parameters, particularly in the case of altered biventricular mechanics at basal evaluation [24]. On the other hand, LV and RV myocardial strain parameters may significantly improve even after a 4-week PR program [23]. These findings suggest the incremental diagnostic and prognostic role of STE analysis over conventional TTE in COPD patients with moderate airflow obstruction.

By highlighting a low effect of COPD on LVEF, our meta-analysis confirmed the limitations and poor sensitivity of LVEF for detecting subclinical LV dysfunction in COPD patients without advanced lung disease. Conversely, given the large effect of COPD on biventricular mechanics revealed by this meta-analysis, the STE methodology showed an incremental diagnostic value over conventional TTE for identifying the early deterioration of myocardial deformation properties of both ventricles. From a clinical point of view, the implementation of conventional TTE with STE analysis of biventricular mechanics may allow clinicians to obtain a more comprehensive assessment of cardiac function in COPD patients with mild-to-moderate airflow limitation.

Concerning cardioprotective treatment, it is known that COPD patients are commonly undertreated with beta blockers due to concerns about the potential occurrence of bronchospasm [51]. The significantly higher heart rate in COPD patients vs. healthy controls, detected by the studies included in this meta-analysis, was likely related to the frequent use of long-acting beta2 agonists and/or undertreatment with beta blockers in COPD individuals. Eminent studies have demonstrated the positive effects of beta blockers in reducing morbidity and mortality in patients with heart failure [52] and previous myocardial infarction [53], and in COPD patients with a CAD history [54]. In agreement with the practical recommendations for the use of beta blockers in COPD patients with CAD, heart failure or arrhythmias [55], a prudent use of cardioselective beta blockers might be considered for initiation and adequate uptitration for those COPD patients with moderate airflow limitation and concomitant impairment in LV- and/or RV-GLS, despite preserved conventional indices of biventricular systolic function.

Despite the beneficial effect of statins on improving lung function and clinical symptoms in COPD patients [56], they are generally underutilized in these individuals, as demonstrated by the results of our meta-analysis, revealing a suboptimal control of LDL cholesterol levels in COPD patients.

### 4.4. Future Directions

Given its ability to detect early impairment in the biventricular myocardial deformation parameters, STE methodology should be considered for implementation in the clinical evaluation of COPD patients without severe airflow obstruction. However, to date, this innovative imaging modality, even if noninvasive and relatively inexpensive, is, overall, underutilized by clinical cardiologists. The main reasons for its underuse are related to incomplete training, time constraints related to image acquisition and analysis [57] and the frequent occurrence of suboptimal echocardiographic windows in COPD patients with consequent poor visualization of the endocardial border of both ventricles and inadequate tracking of the myocardial walls.

Considering the cardioprotective effects of beta blockers and statins, demonstrated in COPD cohorts with relevant cardiovascular comorbidities [54,56], further studies are warranted to evaluate whether the early introduction of these treatments might contribute to improving LV- and/or RV-GLS mechanics in COPD patients also affected by mild-to-moderate airflow limitations.

### 4.5. Study Limitations

The main limitations of the included studies were their monocentric nature, the limited sample size in most studies and the lack of adjusted data for 80% of them. However, our meta-regression analyses excluded any correlation between several moderators and biventricular mechanics.

Another important limitation that potentially affected the generalizability of the research results was the high between-study heterogeneity detected for the studies assessing LV-GLS, RV-GLS and LVEF. This finding was likely related to the inclusion of COPD patients from different countries who were affected by various degrees of COPD and who were examined by different ultrasound machines.

In addition, several clinical, laboratory and echocardiographic parameters were assessed by a limited number of studies. However, it is important to consider that the great majority of COPD patients have high thoracic acoustic impedance [58], leading to poor visualization of the LV and/or RV endocardial borders, with consequent inadequate tracking by STE. Therefore, it is difficult to enroll a consistent number of COPD patients for a prospective assessment of biventricular mechanics.

Moreover, given that the included studies were primarily focused on the assessment of both conventional TTE and innovative STE indices in COPD patients, information concerning both cardiovascular and noncardiovascular comorbidities was scant.

Finally, clinicians should consider that STE analysis has several limitations, such as intervendor variability, dependence on obtaining good image quality, the operator’s experience, the frame rate setting, loading conditions and, finally, extrinsic determinants, particularly anterior chest wall deformity and/or various degrees of pectus excavatum [59,60,61,62]. All these technical factors may contribute to the limited diffusion of this innovative imaging modality among cardiological institutions.

## 5. Conclusions

COPD appears to be associated with the mild attenuation of biventricular mechanics in patients with moderate airflow limitation, despite preserved LVEF and TAPSE on conventional TTE.

Due to its incremental diagnostic and prognostic value, STE analysis may allow clinicians to identify among COPD patients without advanced lung disease those with subclinical myocardial dysfunction and an increased risk of subsequent heart failure and cardiovascular complications early.

Future prospective studies are needed to evaluate if early initiation and/or adequate uptitration of cardioprotective drugs, such as beta blockers and statins, may improve the myocardial deformation properties of both ventricles and/or reduce the future occurrence of adverse cardiovascular events.

## Figures and Tables

**Table 1:** Clinical characteristics and relevant echocardiographic findings of the included studies.

Study Name and Country	Number of Patients	Mean Age (yrs)	Males (%)	Study Design	Ultrasound System	Main Echocardiographic Findings in COPD Patients vs. Healthy Controls
Sabit, R., et al. (2010) [18], United Kingdom	COPD = 36 Controls = 14	COPD = 66.5 Controls = 67	COPD = 52.8 Controls = 64.3	Prospective	GE	↑IVRT, ↔E/A ratio, ↑E/e’ ratio ↔LVEF, ↑aortic PWV ↓LV-GLS, ↓GLSRs ↔sPAP, ↑Tei index, ↓RV-FWLS
Hilde, J.M., et al. (2013) [19], Norway	COPD = 72 Controls = 34	COPD = 63 Controls = 64	COPD = 52.8 Controls = 44.1	Prospective	GE	↔E/A ratio, ↔E/e’ ratio, ↓LVEF ↑RV wall thickness, ↑RV size ↔sPAP, ↑RV-MPI, ↓RV basal s’ ↓TAPSE, ↓RVEF, ↓RV-GLS
Kalaycıoğlu, E., et al. (2015) [20], Turkey	COPD = 125 Controls = 30	COPD = 70 Controls = 67	COPD = 92 Controls = 90	Prospective	NS	↔LV size ↑E/e’ ratio, ↔LVEF ↓LV-GLS, ↓GLSRs ↑sPAP, ↓RV basal s’, ↓TAPSE
Pizarro, C., et al. (2016) [21], Germany	COPD = 51 Controls = 20	COPD = 64.1 Controls = 61.3	COPD = 54.1 Controls = 55	Prospective	GE	↔E/A ratio ↔LVEF ↔sPAP, ↔TAPSE ↓LV-GLS, ↓LV apical septal strain
Xia, Y.J., et al. (2018) [22], China	COPD = 41 Controls = 42	COPD = 49.1 Controls = 48.9	COPD = 53.7 Controls = 61.9	Prospective	Philips	↔LV size ↔E/A ratio, ↔E/e’ ratio, ↔LVEF ↓LV-GLS, ↓LV-GCS, ↓LV-GRS ↓LV peak rotation angle
Kanar, B.G., et al. (2018) [23], Turkey	COPD = 46 Controls = 32	COPD = 60.8 Controls = 58.5	COPD = 61 Controls = 41	Prospective	Philips	↑RV size, ↔RV thickness ↔LVEF, ↔RVEF, ↔RV basal s’ ↑sPAP, ↓TAPSE ↓RV-GLS, ↓RV-FWLS
Nasir, S.A., et al. (2020) [24], India	COPD = 84 Controls = 40	COPD = 63.9 Controls = 61.1	NS	Prospective	GE	↓LVEF ↑sPAP, ↓TAPSE, ↓RV basal s’, ↓RV-FAC ↓RV-GLS
Goedemans, L., et al. (2021) [25], The Netherlands	COPD = 143 Controls = 38	COPD = 69 Controls = 66	COPD = 27 Controls = 53	Retrospective	GE	↔LV size, ↑LAVi ↑E/e’ ratio, ↓LVEF ↑RA size, ↑sPAP, ↓TAPSE ↓LASr, ↓RASr
Cengiz Elçioğlu, B., et al. (2022) [26], Turkey	COPD = 52 Controls = 29	COPD = 60.2 Controls = 57.7	COPD = 100 Controls = 100	Prospective	Philips	↔LV size, ↔LA size, ↔RV size ↔E/A ratio, ↔E/e’ ratio, ↔LVEF ↑sPAP, ↓TAPSE, ↓RV basal s’ ↓LV-GLS, ↓GLSRs
Nguyen Ngoc Dang, H., et al. (2025) [27], Vietnam	COPD = 32 Controls = 37	COPD = 68 Controls = 65	COPD = 91 Controls = 81	Prospective	Philips	↔LVMi, ↔LVEF ↔sPAP, ↔TAPSE ↔LASr, ↓LV-GLS ↓RV-GLS, ↓RV-FWLS

COPD, chronic obstructive pulmonary disease; FAC, fractional area change; FWLS, free wall longitudinal strain; GCS, global circumferential strain; GE, General Electric; GLS, global longitudinal strain; GLSRs, global longitudinal strain rate in systole; GRS, global radial strain; IVRT, isovolumetric relaxation time; LA, left atrial; LASr, left atrial reservoir strain; LAVi, left atrial volume index; LV, left ventricular; LVEF, left ventricular ejection fraction; LVMi, left ventricular mass index; MPI, myocardial performance index; NS, not specified; PWV, pulse wave velocity; RA, right atrial; RASr, right atrial reservoir strain; RV, right ventricular; RVEF, right ventricular ejection fraction; sPAP, systolic pulmonary artery pressure; TAPSE, tricuspid annular plane systolic excursion.

**Table 2:** Demographic, anthropometric, clinical, hemodynamic, spirometric and biochemical parameters collected at baseline by the included studies in COPD patients and healthy controls.

	Number of Studies for Parameters Assessed (%)	Sample Size COPD vs. Controls	COPD	Controls	*p*-Value
**Demographics**					
Age (yrs)	10 (100)	682 vs. 316	63.5 (49.1−70)	61.6 (48.9−67)	<0.05
Males (%)	9 (90)	598 vs. 276	64.9 (27−100)	65.6 (41−100)	NS
**Anthropometrics**					
BSA (m^2^)	4 (40)	331 vs. 149	1.75 (1.52−2)	1.77 (1.56−1.9)	NS
BMI (Kg/m^2^)	7 (70)	455 vs. 212	25.1 (20.3−28.2)	25.6 (21.3−27.9)	<0.05
**Cardiovascular risk factors**					
Hypertension (%)	3 (30)	319 vs. 88	58.2 (41−76)	30 (0−50)	<0.05
Current smoking (%)	4 (40)	391 vs. 122	32 (20−43)	11.2 (0−23)	<0.05
Pack—years of smoking	5 (50)	316 vs. 135	41.9 (30−52)	10.9 (0−30)	<0.05
Type 2 diabetes (%)	3 (30)	319 vs. 88	16.4 (14−21)	5.3 (0−10)	<0.05
Dyslipidemia (%)	3 (30)	266 vs. 92	44 (38.2−55)	17.5 (0−35)	<0.05
**Cardiovascular comorbidities**					
CAD (%)	2 (20)	194 vs. 58	39.1 (28.2−50)	5 (0−10)	<0.05
**Noncardiovascular comorbidities**					
OSAS (%)	1 (10)	143 vs. 38	4	0	NS
**Hemodynamics**					
Sinus rhythm (%)	10 (100)	682 vs. 316	89.9 (0−100)	100	<0.05
AF (%)	10 (100)	682 vs. 316	10.1 (0−100)	0	<0.05
Heart rate (bpm)	6 (60)	371 vs. 172	80.3 (71.4−96.5)	72.2 (60.3−80.4)	<0.05
SBP (mmHg)	6 (60)	406 vs. 212	126 (114−139)	121.6 (111−133.9)	<0.05
DBP (mmHg)	6 (60)	406 vs. 212	74.5 (69−83.2)	75 (68.1−80.9)	NS
**COPD severity**					
GOLD stage	6 (60)	428	1.9 (1.5−2.5)	/	/
BODE index	3 (30)	255	2.5 (2−3)	/	/
**Pulmonary function tests**					
FEV1 (% predicted)	6 (60)	371 vs. 172	51.9 (45−60.1)	97.3 (89−103.6)	<0.05
FVC (% predicted)	3 (30)	233 vs. 78	77.4 (74.6−81.7)	98.3 (85.4−105)	<0.05
FEV1/FVC (%)	7 (70)	455 vs. 212	56 (49−60.5)	82.6 (76−96.8)	<0.05
RV (% predicted)	2 (20)	123 vs. 54	181.5 (171−192)	115.2 (111.4−119)	<0.05
DLCO (% predicted)	2 (20)	123 vs. 54	52.7 (48.5−57)	89.1 (78.3−100)	<0.05
6MWD (m)	2 (20)	156 vs. 74	379 (343−415)	494.5 (489−500)	<0.05
**Blood gas analysis**					
SaO2 (%)	4 (40)	289 vs. 126	93.6 (92−96)	96.1 (96−96.3)	<0.05
PaO2 (mmHg)	4 (40)	289 vs. 126	69.4 (66.4−73.5)	77.7 (63.7−92)	<0.05
PaCO2 (mmHg)	4 (40)	289 vs. 126	37.9 (35.7−40)	35 (34.1−36)	<0.05
**Biochemical parameters**					
Hemoglobin (g/dL)	4 (40)	360 vs. 130	14.2 (13.2−15.8)	14.2 (12.6−16.2)	NS
Creatinine (g/dL)	2 (20)	184 vs. 80	0.97 (0.75−1.18)	0.80 (0.72−0.88)	<0.05
Fasting glucose (mg/dL)	2 (20)	161 vs. 44	99.6 (95.6−103.6)	97.1 (94.1−100.1)	<0.05
LDL cholesterol (mg/dL)	2 (20)	193 vs. 81	129.8 (119.1−140.5)	124.9 (124−125.9)	<0.05
NT-proBNP (pg/mL)	2 (20)	123 vs. 54	268.4 (83.7−453.2)	87.7 (78.6−96.8)	<0.05
**Nonrespiratory medical treatment**					
ACE-i/ARBs (%)	2 (20)	268 vs. 68	45 (25−65)	12 (0−24)	<0.05
CCB (%)	2 (20)	268 vs. 68	14.5 (14−15)	8.5 (0−17)	<0.05
BB (%)	1 (10)	125 vs. 30	4	10	NS
Statins (%)	1 (10)	125 vs. 30	17	13	NS
Oral hypoglycemic agents (%)	1 (10)	125 vs. 30	11	3	NS
Insulin (%)	1 (10)	125 vs. 30	4	3	NS
**Respiratory medical treatment**					
Home oxygen therapy (%)	1 (10)	51	20	/	/
Short-acting beta2-agonist (%)	1 (10)	143	15	/	/
Long-acting beta2-agonist (%)	2 (20)	194	63.8 (57−70.6)	/	/
Long-acting anticholinergic (%)	1 (10)	51	60	/	/
Inhaled glucocorticoid (%)	2 (20)	194	49 (34.1−64)	/	/
Systemic glucocorticoid (%)	1 (10)	51	4.7	/	/
PDE-4 inhibitor (%)	1 (10)	51	17.6	/	/

Data are expressed as the median and interquartile range. 6MWD, 6-min walk distance; ACE-i, angiotensin-converting-enzyme inhibitors; AF, atrial fibrillation; ARBs, angiotensin receptor blockers; BB, beta blockers; BMI, body mass index; BODE, Body-Mass Index, Airflow Obstruction, Dyspnea and Exercise; BSA; body surface area; CAD, coronary artery disease; CCB, calcium channel blockers; COPD, chronic obstructive pulmonary disease; DBP, diastolic blood pressure; DLCO, diffusing capacity of the lungs for carbon monoxide; FEV1, forced expiratory volume in the first second; FVC, forced vital capacity; GOLD, Global Initiative for Obstructive Lung Disease; LDL, low-density lipoprotein; NS, statistically non-significant; NT-proBNP, N-terminal pro B-type natriuretic peptide; PaO2, arterial partial pressure of oxygen; OSAS, obstructive sleep apnea syndrome; PaCO2, partial pressure of carbon dioxide in arterial blood; PDE-4, phosphodiesterase-4; RV, residual volume; SaO2, arterial oxygen saturation; SBP, systolic blood pressure.

**Table 3:** Echocardiographic parameters measured by traditional transthoracic echocardiography and speckle tracking echocardiography in COPD patients and healthy controls.

Echocardiographic Parameters	Number of Studies for Parameters Assessed (%)	Sample Size COPD vs. Controls	COPD	Controls	*p*-Value
**TTE parameters**					
IVS thickness (mm)	2 (20)	177 vs. 59	10.5 (10−11.1)	10.4 (9.9−11)	NS
LV-PW thickness (mm)	2 (20)	177 vs. 59	10.2 (9.8−10.6)	10 (9.8−10.2)	<0.05
LV-EDD (mm)	3 (30)	218 vs. 101	46.9 (45.8−48)	47.2 (46.3−48)	<0.04
LV-ESD (mm)	3 (30)	218 vs. 101	29.6 (28.1−32)	29.4 (27.7−32)	NS
RWT	2 (20)	177 vs. 59	0.44 (0.41−0.46)	0.42 (0.41−0.44)	<0.05
LVMi (g/m^2^)	2 (20)	68 vs. 51	95.5 (86.5−104.5)	93.9 (84.1−103.8)	NS
LVEF (%)	10 (100)	682 vs. 316	60 (50−67.8)	62.7 (59.4−69.1)	<0.05
SV (mL)	2 (20)	113 vs. 76	58.7 (47.2−70.2)	58.1 (47.8−68.4)	NS
CO (L/min)	2 (20)	113 vs. 76	4.4 (3.6−5.2)	4.6 (3.6−5.6)	NS
E/A ratio	4 (40)	201 vs. 119	1.09 (0.8−1.43)	1.14 (0.9−1.51)	NS
E/e’ ratio	6 (60)	469 vs. 187	9.7 (5.8−17)	7.7 (6.1−11)	<0.05
LAVi (ml/m^2^)	2 (20)	215 vs. 72	35.5 (24−47)	22 (21−23)	<0.05
RV-EDD (mm)	4 (40)	206 vs. 109	30.5 (24−38.1)	26 (24−27.6)	<0.05
TDI RV basal s’ (cm/s)	6 (60)	415 vs. 179	11.6 (10−12.9)	13.3 (12−15.2)	<0.05
RVEF (%)	2 (20)	118 vs. 66	52.4 (50−54.8)	57.1 (56.3−58)	<0.05
TAPSE (mm)	7 (70)	533 vs. 226	19.3 (16.6−23)	22 (19.7−26)	<0.05
sPAP (mmHg)	9 (90)	641 vs. 274	32.3 (22.9−46.7)	23.2 (18−30)	<0.05
**STE parameters**					
LV-GLS (%)	6 (60)	337 vs. 172	17.1 (13.9−18.9)	19.9 (17.1−22.3)	<0.05
LV-GLSRs (s^−1^)	3 (30)	213 vs. 73	1.28 (0.9−1.6)	1.34 (1−1.7)	NS
LV-GCS (%)	1 (10)	41 vs. 42	18.9 (16.3−21.5)	19.2 (16.6−21.8)	NS
LV-GRS (%)	1 (10)	41 vs. 42	25.9 (20.4−31.4)	26.5 (21−32)	NS
RV-GLS (%)	5 (50)	270 vs. 157	19.5 (14.6−22)	25.4 (18.3−31)	<0.05
RV-FWLS (%)	2 (20)	78 vs. 69	17.3 (16.5−18.1)	24.6 (21.4−27.9)	<0.05
LASr (%)	2 (20)	175 vs. 75	21.8 (14.2−29.5)	30.6 (30.2−31.1)	<0.05
RASr (%)	1 (10)	143 vs. 38	15.3 (9−25.1)	42.8 (33.7−48.3)	<0.05

Data are expressed as the median and interquartile range. CO, cardiac output; COPD, chronic obstructive pulmonary disease; EDD, end-diastolic diameter; ESD, end-systolic diameter; FWLS, free wall longitudinal strain; GCS, global circumferential strain; GLS, global longitudinal strain; GLSRs, global longitudinal strain rate in systole; GRS, global radial strain; IVS, interventricular septum; LASr, left atrial reservoir strain; LAVi, left atrial volume index; LV, left ventricular; LVEF, left ventricular ejection fraction; LVMi, left ventricular mass index; NS, statistically non-significant; PW, posterior wall; RASr, right atrial reservoir strain; RV, right ventricular; RVEF, right ventricular ejection fraction; RWT, relative wall thickness; sPAP, systolic pulmonary artery pressure; STE, speckle tracking echocardiography; SV, stroke volume; TAPSE, tricuspid annular plane systolic excursion; TDI, tissue Doppler imaging; TTE, transthoracic echocardiography.

**Table 4:** Quality Assessment of Case-Control Studies [18,19,20,21,22,23,24,25,26,27]. Q1–Q12 items are accessible from the URL: https://www.nhlbi.nih.gov/health-topics/study-quality-assessment-tools (accessed on 30 April 2025). NS, not specified.

NIH Quality Assessment Tool of Case-Control Studies Criteria Met													
Study Name	Q1	Q2	Q3	Q4	Q5	Q6	Q7	Q8	Q9	Q10	Q11	Q12	Quality
Sabit R. et al. [18]	Yes	Yes	Yes	NS	Yes	Yes	NS	Yes	Yes	Yes	Yes	No	9 (Good)
Hilde J.M. et al. [19]	Yes	Yes	No	Yes	Yes	Yes	NS	Yes	Yes	Yes	Yes	Yes	10 (Good)
Kalaycıoğlu E. et al. [20]	Yes	Yes	No	Yes	Yes	Yes	NS	Yes	Yes	Yes	NS	No	8 (Fair)
Pizarro C. et al. [21]	Yes	Yes	No	Yes	Yes	Yes	Yes	Yes	Yes	Yes	Yes	No	10 (Good)
Xia Y.J. et al. [22]	Yes	Yes	No	Yes	Yes	Yes	NS	Yes	Yes	Yes	NS	No	8 (Fair)
Kanar B.G. et al. [23]	Yes	Yes	Yes	NS	Yes	Yes	NS	Yes	Yes	Yes	NS	No	8 (Fair)
Nasir S.A. et al. [24]	Yes	Yes	No	Yes	Yes	Yes	NS	Yes	Yes	Yes	NS	No	8 (Fair)
Goedemans L. et al. [25]	Yes	Yes	No	Yes	Yes	Yes	NS	Yes	Yes	Yes	NS	Yes	9 (Good)
Cengiz Elçioğlu B. et al. [26]	Yes	Yes	No	NS	Yes	Yes	NS	Yes	Yes	Yes	NS	No	7 (Fair)
Nguyen Ngoc Dang H. et al. [27]	Yes	Yes	No	Yes	Yes	Yes	NS	Yes	Yes	Yes	NS	No	8 (Fair)

**Table 5:** Meta-regression analysis performed to assess the impact of potential confounders on LV-GLS. BMI, body mass index; GLS, global longitudinal strain; LV, left ventricular; SBP, systolic blood pressure.

	Coefficient	Standard Error	95%CI Lower	95%CI Upper	*p*-Value
Intercept	−3.7559	8.9379	−21.274	13.7621	0.67
Age	−0.1431	0.0978	−0.3348	0.0485	0.14
BMI	−0.0659	0.1855	−0.4296	0.2978	0.72
SBP	0.1057	0.0866	−0.0639	0.2754	0.22

**Table 6:** Meta-regression analysis performed to assess the impact of potential confounders on RV-GLS. GE, General Electric; GLS, global longitudinal strain; RV, right ventricular; sPAP, systolic pulmonary artery pressure.

	Coefficient	Standard Error	95%CI Lower	95%CI Upper	*p*-Value
Intercept	−0.3641	3.9875	−8.1795	7.4512	0.93
NonGE ultrasound machine	0.0817	1.0647	−2.0051	2.1685	0.94
sPAP	−0.0402	0.1424	−0.3194	0.239	0.78

## Data Availability

Data extracted from the included studies will be publicly available on Zenodo (https://zenodo.org, accessed on 30 April 2025), pending acceptance by the journal.

## References

[1] Celli B.R., Fabbri L.M., Aaron S.D., Agusti A., Brook R., Criner G.J., Franssen F.M.E., Humbert M., Hurst J.R., O’Donnell D. (2021). An Updated Definition and Severity Classification of Chronic Obstructive Pulmonary Disease Exacerbations: The Rome Proposal. Am. J. Respir. Crit. Care Med..

[2] Adeloye D., Chua S., Lee C., Basquill C., Papana A., Theodoratou E., Nair H., Gasevic D., Sridhar D., Campbell H. (2015). Global and regional estimates of COPD prevalence: Systematic review and meta-analysis. J. Glob. Health.

[3] Liu X., Chen Z., Li S., Xu S. (2021). Association of Chronic Obstructive Pulmonary Disease With Arrhythmia Risks: A Systematic Review and Meta-Analysis. Front. Cardiovasc. Med..

[4] Zheng Y., Hu Z., Seery S., Li C., Yang J., Wang W., Qi Y., Shao C., Fu Y., Xiao H. (2024). Global Insights into Chronic Obstructive Pulmonary Disease and Coronary Artery Disease: A Systematic Review and Meta-Analysis of 6,400,000 Patients. Rev. Cardiovasc. Med..

[5] Papaporfyriou A., Bartziokas K., Gompelmann D., Idzko M., Fouka E., Zaneli S., Bakakos P., Loukides S., Papaioannou A.I. (2023). Cardiovascular Diseases in COPD: From Diagnosis and Prevalence to Therapy. Life.

[6] Brassington K., Selemidis S., Bozinovski S., Vlahos R. (2019). New frontiers in the treatment of comorbid cardiovascular disease in chronic obstructive pulmonary disease. Clin. Sci..

[7] Macchia A., Rodriguez Moncalvo J.J., Kleinert M., Comignani P.D., Gimeno G., Arakaki D., Laffaye N., Fuselli J.J., Massolin H.P., Gambarte J. (2012). Unrecognised ventricular dysfunction in COPD. Eur. Respir. J..

[8] Potter E., Marwick T.H. (2018). Assessment of Left Ventricular Function by Echocardiography: The Case for Routinely Adding Global Longitudinal Strain to Ejection Fraction. JACC Cardiovasc. Imaging..

[9] Grenne B., Eek C., Sjøli B., Dahlslett T., Uchto M., Hol P.K., Skulstad H., Smiseth O.A., Edvardsen T., Brunvand H. (2010). Acute coronary occlusion in non-ST-elevation acute coronary syndrome: Outcome and early identification by strain echocardiography. Heart.

[10] Sonaglioni A., Albini A., Fossile E., Pessi M.A., Nicolosi G.L., Lombardo M., Anzà C., Ambrosio G. (2020). Speckle-Tracking Echocardiography for Cardioncological Evaluation in Bevacizumab-Treated Colorectal Cancer Patients. Cardiovasc. Toxicol..

[11] Purwowiyoto S.L., Halomoan R. (2022). Highlighting the role of global longitudinal strain assessment in valvular heart disease. Egypt. Heart J..

[12] Moher D., Liberati A., Tetzlaff J., Altman D.G., PRISMA Group (2009). Preferred reporting items for systematic reviews and meta-analyses: The PRISMA Statement. Open Med..

[13] Global Initiative for Chronic Obstructive Lung Disease [Homepage on the Internet]. Global Strategy for Prevention; Diagnosis and Management of COPD: 2025 Report. https://goldcopd.org/2025-gold-report/.

[14] Humbert M., Kovacs G., Hoeper M.M., Badagliacca R., Berger R.M.F., Brida M., Carlsen J., Coats A.J.S., Escribano-Subias P., Ferrari P. (2023). 2022 ESC/ERS Guidelines for the diagnosis and treatment of pulmonary hypertension. Eur. Respir. J..

[15] Celli B.R., Cote C.G., Marin J.M., Casanova C., Montes de Oca M., Mendez R.A., Pinto Plata V., Cabral H.J. (2004). The body-mass index, airflow obstruction, dyspnea, and exercise capacity index in chronic obstructive pulmonary disease. N. Engl. J. Med..

[16] Ma L.L., Wang Y.Y., Yang Z.H., Huang D., Weng H., Zeng X.T. (2020). Methodological quality (risk of bias) assessment tools for primary and secondary medical studies: What are they and which is better?. Mil. Med. Res..

[17] McHugh M.L. (2012). Interrater reliability: The kappa statistic. Biochem. Med..

[18] Sabit R., Bolton C.E., Fraser A.G., Edwards J.M., Edwards P.H., Ionescu A.A., Cockcroft J.R., Shale D.J. (2010). Sub-clinical left and right ventricular dysfunction in patients with COPD. Respir. Med..

[19] Hilde J.M., Skjørten I., Grøtta O.J., Hansteen V., Melsom M.N., Hisdal J., Humerfelt S., Steine K. (2013). Right ventricular dysfunction and remodeling in chronic obstructive pulmonary disease without pulmonary hypertension. J. Am. Coll. Cardiol..

[20] Kalaycıoğlu E., Gökdeniz T., Aykan A.Ç., Hatem E., Gürsoy M.O., Toksoy F., Dursun I., Çelik S. (2015). Evaluation of Left Ventricular Function and its Relationship With Multidimensional Grading System (BODE Index) in Patients with COPD. COPD.

[21] Pizarro C., van Essen F., Linnhoff F., Schueler R., Hammerstingl C., Nickenig G., Skowasch D., Weber M. (2016). Speckle tracking echocardiography in chronic obstructive pulmonary disease and overlapping obstructive sleep apnea. Int. J. Chron. Obstruct. Pulmon. Dis..

[22] Xia Y.J., Sun H.Y., Jiang L., Liu B., Wang Y.Y. (2018). Evaluation of the effects of right ventricular pressure load on left ventricular myocardial mechanics in patients with chronic obstructive pulmonary disease by ultrasound speckle tracking imaging. Eur. Rev. Med. Pharmacol. Sci..

[23] Kanar B.G., Ozmen I., Yildirim E.O., Ozturk M., Sunbul M. (2018). Right Ventricular Functional Improvement after Pulmonary Rehabilitation Program in Patients with COPD Determined by Speckle Tracking Echocardiography. Arq. Bras. Cardiol..

[24] Nasir S.A., Singh S., Fotedar M., Chaudhari S.K., Sethi K.K. (2020). Echocardiographic Evaluation of Right Ventricular Function and its Role in the Prognosis of Chronic Obstructive Pulmonary Disease. J. Cardiovasc. Echogr..

[25] Goedemans L., Leung M., van der Bijl P., Abou R., Vo N.M., Ajmone Marsan N., Delgado V., Bax J.J. (2021). Influence of Chronic Obstructive Pulmonary Disease on Atrial Mechanics by Speckle Tracking Echocardiography in Patients With Atrial Fibrillation. Am. J. Cardiol..

[26] Cengiz Elçioğlu B., Kamat S., Yurdakul S., Şahin Ş.T., Sarper A., Yıldız P., Aytekin S. (2022). Assessment of subclinical left ventricular systolic dysfunction and structural changes in patients with chronic obstructive pulmonary disease. Intern. Med. J..

[27] Nguyen Ngoc Dang H., Viet Luong T., Thi Y Nguyen N., Khanh Tran H., Thi Nguyen Tran H., Minh Vu H., Van Ho T., Thi Minh Vo N., Thien Tran T., Song Do T. (2025). Assessment of the right ventricular strain; left ventricular strain and left atrial strain using speckle tracking echocardiography in patients with chronic obstructive pulmonary disease. BMJ Open Respir. Res..

[28] Levey A.S., Stevens L.A., Schmid C.H., Zhang Y.L., Castro A.F., Feldman H.I., Kusek J.W., Eggers P., Van Lente F., Greene T. (2009). A new equation to estimate glomerular filtration rate. Ann. Intern. Med..

[29] Nagueh S.F., Smiseth O.A., Appleton C.P., Byrd B.F., Dokainish H., Edvardsen T., Flachskampf F.A., Gillebert T.C., Klein A.L., Lancellotti P. (2016). Recommendations for the Evaluation of Left Ventricular Diastolic Function by Echocardiography: An Update from the American Society of Echocardiography and the European Association of Cardiovascular Imaging. Eur. Heart J. Cardiovasc. Imaging..

[30] Galderisi M., Cosyns B., Edvardsen T., Cardim N., Delgado V., Di Salvo G., Donal E., Sade L.E., Ernande L., Garbi M. (2017). Standardization of adult transthoracic echocardiography reporting in agreement with recent chamber quantification; diastolic function; and heart valve disease recommendations: An expert consensus document of the European Association of Cardiovascular Imaging. Eur. Heart J. Cardiovasc. Imaging..

[31] Muraru D., Onciul S., Peluso D., Soriani N., Cucchini U., Aruta P., Romeo G., Cavalli G., Iliceto S., Badano L.P. (2016). Sex- and Method-Specific Reference Values for Right Ventricular Strain by 2-Dimensional Speckle-Tracking Echocardiography. Circ. Cardiovasc. Imaging..

[32] Hung C.L., Gonçalves A., Shah A.M., Cheng S., Kitzman D., Solomon S.D. (2017). Age- and Sex-Related Influences on Left Ventricular Mechanics in Elderly Individuals Free of Prevalent Heart Failure: The ARIC Study (Atherosclerosis Risk in Communities). Circ. Cardiovasc. Imaging.

[33] Soufi Taleb Bendiab N., Meziane-Tani A., Ouabdesselam S., Methia N., Latreche S., Henaoui L., Monsuez J.J., Benkhedda S. (2017). Factors associated with global longitudinal strain decline in hypertensive patients with normal left ventricular ejection fraction. Eur. J. Prev. Cardiol..

[34] Watanabe Y., Tajiri K., Suzuki A., Nagata H., Kojima M. (2020). Influence of cigarette smoking on biventricular systolic function independent of respiratory function: A cross-sectional study. BMC Cardiovasc. Disord..

[35] Sin D.D., Doiron D., Agusti A., Anzueto A., Barnes P.J., Celli B.R., Criner G.J., Halpin D., Han M.K., Martinez F.J. (2023). Air pollution and COPD: GOLD 2023 committee report. Eur. Respir. J..

[36] Badesch D.B., Champion H.C., Gomez Sanchez M.A., Hoeper M.M., Loyd J.E., Manes A., McGoon M., Naeije R., Olschewski H., Oudiz R.J. (2009). Diagnosis and assessment of pulmonary arterial hypertension. J. Am. Coll. Cardiol..

[37] Hermann E.A., Sun Y., Hoffman E.A., Allen N.B., Ambale-Venkatesh B., Bluemke D.A., Carr J.J., Kawut S.M., Prince M.R., Shah S.J. (2024). Lung structure and longitudinal change in cardiac structure and function: The MESA COPD Study. Eur. Respir. J..

[38] Hilde J.M., Skjørten I., Hansteen V., Melsom M.N., Hisdal J., Humerfelt S., Steine K. (2013). Haemodynamic responses to exercise in patients with COPD. Eur. Respir. J..

[39] Tello K., Ghofrani H.A., Heinze C., Krueger K., Naeije R., Raubach C., Seeger W., Sommer N., Gall H., Richter M.J. (2019). A simple echocardiographic estimate of right ventricular-arterial coupling to assess severity and outcome in pulmonary hypertension on chronic lung disease. Eur. Respir. J..

[40] He Q., Lin Y., Zhu Y., Gao L., Ji M., Zhang L., Xie M., Li Y. (2023). Clinical Usefulness of Right Ventricle-Pulmonary Artery Coupling in Cardiovascular Disease. J. Clin. Med..

[41] Sin D.D., Man S.F. (2005). Chronic obstructive pulmonary disease: A novel risk factor for cardiovascular disease. Can. J. Physiol. Pharmacol..

[42] Cavaillès A., Brinchault-Rabin G., Dixmier A., Goupil F., Gut-Gobert C., Marchand-Adam S., Meurice J.C., Morel H., Person-Tacnet C., Leroyer C. (2013). Comorbidities of COPD. Eur. Respir. Rev..

[43] Ozben B., Eryüksel E., Tanrikulu A.M., Papila-Topal N., Celikel T., Başaran Y. (2010). Acute exacerbation impairs endothelial function in patients with chronic obstructive pulmonary disease. Turk. Kardiyol. Dern. Ars..

[44] Maclay J.D., MacNee W. (2013). Cardiovascular disease in COPD: Mechanisms. Chest.

[45] Ukena C., Mahfoud F., Kindermann M., Kindermann I., Bals R., Voors A.A., van Veldhuisen D.J., Böhm M. (2010). The cardiopulmonary continuum systemic inflammation as ’common soil’ of heart and lung disease. Int. J. Cardiol..

[46] Seta Y., Shan K., Bozkurt B., Oral H., Mann D.L. (1996). Basic mechanisms in heart failure: The cytokine hypothesis. J. Card. Fail..

[47] Sonaglioni A., Nicolosi G.L., Trevisan R., Granato A., Zompatori M., Lombardo M. (2022). Modified Haller index validation and correlation with left ventricular strain in a cohort of subjects with obesity and without overt heart disease. Intern. Emerg. Med..

[48] Sonaglioni A., Nicolosi G.L., Granato A., Lombardo M., Anzà C., Ambrosio G. (2021). Reduced Myocardial Strain Parameters in Subjects With Pectus Excavatum: Impaired Myocardial Function or Methodological Limitations Due to Chest Deformity?. Semin. Thorac. Cardiovasc. Surg..

[49] Niki K., Sugawara M., Kayanuma H., Takamisawa I., Watanabe H., Mahara K., Sumiyoshi T., Ida T., Takanashi S., Tomoike H. (2017). Associations of increased arterial stiffness with left ventricular ejection performance and right ventricular systolic pressure in mitral regurgitation before and after surgery: Wave intensity analysis. Int. J. Cardiol. Heart Vasc..

[50] Chaouat A., Naeije R., Weitzenblum E. (2008). Pulmonary hypertension in COPD. Eur. Respir. J..

[51] van der Woude H.J., Zaagsma J., Postma D.S., Winter T.H., van Hulst M., Aalbers R. (2005). Detrimental effects of beta-blockers in COPD: A concern for nonselective beta-blockers. Chest.

[52] Hjalmarson A., Goldstein S., Fagerberg B., Wedel H., Waagstein F., Kjekshus J., Wikstrand J., El Allaf D., Vítovec J., Aldershvile J. (2000). Effects of controlled-release metoprolol on total mortality; hospitalizations; and well-being in patients with heart failure: The Metoprolol CR/XL Randomized Intervention Trial in congestive heart failure (MERIT-HF). MERIT-HF Study Group. JAMA.

[53] Gottlieb S.S., McCarter R.J., Vogel R.A. (1998). Effect of beta-blockade on mortality among high-risk and low-risk patients after myocardial infarction. N. Engl. J. Med..

[54] Andell P., Erlinge D., Smith J.G., Sundström J., Lindahl B., James S., Koul S. (2015). β-blocker use and mortality in COPD patients after myocardial infarction: A Swedish nationwide observational study. J. Am. Heart Assoc..

[55] Wade C., Wells J.M. (2020). Practical recommendations for the use of beta-blockers in chronic obstructive pulmonary disease. Expert Rev. Respir. Med..

[56] Chen X., Hu F., Chai F., Chen X. (2023). Effect of statins on pulmonary function in patients with chronic obstructive pulmonary disease: A systematic review and meta-analysis of randomized controlled trials. J. Thorac. Dis..

[57] Sade L.E., Joshi S.S., Cameli M., Cosyns B., Delgado V., Donal E., Edvardsen T., Carvalho R.F., Manka R., Podlesnikar T. (2023). Current clinical use of speckle-tracking strain imaging: Insights from a worldwide survey from the European Association of Cardiovascular Imaging (EACVI). Eur. Heart J. Cardiovasc. Imaging..

[58] Rao A., Huynh E., Royston T.J., Kornblith A., Roy S. (2019). Acoustic Methods for Pulmonary Diagnosis. IEEE Rev. Biomed. Eng..

[59] Farsalinos K.E., Daraban A.M., Ünlü S., Thomas J.D., Badano L.P., Voigt J.U. (2015). Head-to-Head Comparison of Global Longitudinal Strain Measurements among Nine Different Vendors: The EACVI/ASE Inter-Vendor Comparison Study. J. Am. Soc. Echocardiogr..

[60] Negishi T., Negishi K., Thavendiranathan P., Cho G.Y., Popescu B.A., Vinereanu D., Kurosawa K., Penicka M., Marwick T.H., SUCCOUR Investigators (2017). Effect of Experience and Training on the Concordance and Precision of Strain Measurements. JACC Cardiovasc. Imaging.

[61] Rösner A., Barbosa D., Aarsæther E., Kjønås D., Schirmer H., D’hooge J. (2015). The influence of frame rate on two-dimensional speckle-tracking strain measurements: A study on silico-simulated models and images recorded in patients. Eur. Heart J. Cardiovasc. Imaging.

[62] Sonaglioni A., Fagiani V., Nicolosi G.L., Lombardo M. (2024). The influence of pectus excavatum on biventricular mechanics: A systematic review and meta-analysis. Minerva Cardiol. Angiol..

